# Towards Haptic-Based Dual-Arm Manipulation

**DOI:** 10.3390/s23010376

**Published:** 2022-12-29

**Authors:** Sri Harsha Turlapati, Domenico Campolo

**Affiliations:** School of Mechanical and Aerospace Engineering, Nanyang Technological University, Singapore 639798, Singapore

**Keywords:** object pose tracking, haptic, active manipulation, dual-arm manipulation, object localization, object manipulation, haptic manipulation

## Abstract

Vision is the main component of current robotics systems that is used for manipulating objects. However, solely relying on vision for hand−object pose tracking faces challenges such as occlusions and objects moving out of view during robotic manipulation. In this work, we show that object kinematics can be inferred from local haptic feedback at the robot−object contact points, combined with robot kinematics information given an initial vision estimate of the object pose. A planar, dual-arm, teleoperated robotic setup was built to manipulate an object with hands shaped like circular discs. The robot hands were built with rubber cladding to allow for rolling contact without slipping. During stable grasping by the dual arm robot, under quasi-static conditions, the surface of the robot hand and object at the contact interface is defined by local geometric constraints. This allows one to define a relation between object orientation and robot hand orientation. With rolling contact, the displacement of the contact point on the object surface and the hand surface must be equal and opposite. This information, coupled with robot kinematics, allows one to compute the displacement of the object from its initial location. The mathematical formulation of the geometric constraints between robot hand and object is detailed. This is followed by the methodology in acquiring data from experiments to compute object kinematics. The sensors used in the experiments, along with calibration procedures, are presented before computing the object kinematics from recorded haptic feedback. Results comparing object kinematics obtained purely from vision and from haptics are presented to validate our method, along with the future ideas for perception via haptic manipulation.

## 1. Approaches to Dexterous Robotic Manipulation

Dexterous manipulation of an object is the ability to change the object’s position and orientation [1] while grasping it in the hand—an ability that has let humans use tools and arguably develop a superior brain even [2]. Robotics manipulation research has attempted to mimic this human ability by building robot hands with the aim of achieving similar dexterity artificially for both industrial [3] and home [4,5,6] applications.

Despite impressive results in robotic manipulation showcased for warehouse operations [7], robots still fall short of human-like levels of speed and reliability. Most robot solutions use only one robotic hand with limited gripper orientation [8]. This greatly limits speed, manipulative payload and dexterity, as humans can use two arms.

With dual-arm manipulation, state estimation and control are determined by the contact type between the robot hands and object, i.e., (i) point contact (with and without friction), (ii) sliding contact and (iii) rolling contact [9,10]. With most practical situations having friction between hand and object, point contacts are problematic if the objective is to move the object dexterously without break of contact. For this reason, rolling manipulation increases the dexterity with the hand boundary, allowing for dexterous motion of the object [2,11].

The forward kinematics of rolling-contact dual-arm manipulation was rigorously investigated in the late 1980s [12,13] using geometry. The rolling constraints were used to derive the relation between robot velocities and object velocities, and a joint torque controller was developed to simulate the contact forces due to rolling contact. Going a step further to kinematic constraints, a dynamical closed loop controller was developed in [14,15,16]—where the only knowledge needed was sensing of finger−robot states and kinematic parameters in a finger model. This approach was called *blind grasping* and has spawned more research in recent years [17], even with tackling challenges such as unknown object mass, shape, Coriolis terms and hand/object kinematics [18]. Although it is desirable to perform manipulation with as little information as possible as reasoned in [15], it is advantageous in practical situations to choose to use the information which is readily available—robot hand geometry, robot parameters, etc. For instance, contact-point sensing using hand geometry is useful in determining object pose during manipulation in real time, as we will show in this work. Discarding this haptic information could be disadvantageous and would need alternative approaches to manipulation control. In fact, much of the robotic manipulation literature focuses on using vision to determine object pose information, as we discuss next.

Much work regarding vision-based robotic grasping and manipulation for parallel jaw grippers mounted on a single robot arm has been reported in the surveys by [8,19,20]. Such vision-based approaches present fundamental limitations when it comes to dexterous manipulation tasks requiring more accurate and controlled contact interactions, such as object reorientation, object insertion or almost any kind of object use [21]. Many practical applications, especially in home settings, would require robots to have *semi-precise* or *gentle* placement of components, which would require contact information about the object pose and haptic feedback during manipulation. This further motivates the case for using haptic feedback during robot manipulation for perception and control.

### Contributions and Organization of Paper

In this work, we propose a simple approach using the concept of the wrench axis, which allows for contact sensing. Tactile sensors [22] are usually placed on the robot’s end-effector tips, which enable sensing the point of contact effectively. In this work, we chose to place the force sensors on the robot wrists instead. This choice allows for a measurement of a wrench, which indirectly contains contact point information when viewed with robot hand geometry (which is information that should be available to every robotics engineer). We present a method to estimate online the object pose using (i) an initial kinematic estimate obtained from vision and (ii) real-time haptic feedback from the hands in a dual-arm robotic manipulation setting. We also show that we can reconstruct the normal and tangential forces at contact using the geometry at the contact frame.

The rest of the paper is organized as follows. In Section 2, we present a haptic method to determine contact frame using the wrench axis (estimated using force/torque measurements alone) for a circular robot hand. This haptic method is one of the main contributions of this work. To validate the contact frame information obtained, we demonstrate the computation of object orientation under rolling contact (without slippage) by tracking the contact point on the robot hand. With promising results for estimating kinematics from haptic feedback for a single hand, we present the forward kinematics for dual-arm robotic manipulation in Section 3. The second contribution of our work is the algorithm to compute object position and orientation at any time using (i) information about initial hand−object kinematics and (ii) hand kinematics and haptic feedback at any time. This is presented in Section 4 along with the method to fuse kinematic estimates from two hands. The experimental validation of the proposed algorithm is presented in Section 5, which reports on a human operator performing a haptic demonstration of dual-arm manipulation using a teleoperated robot setup. The object position and orientation were tracked using vision (with a high accuracy motion capture system) and also computed using our proposed algorithm, and the results are compared to validate our method. Finally, the conclusions are presented along with sensor calibration reported in Appendix A.1 and numerical details of the noise present in sensed wrenches in Appendix A.2.

## 2. Haptic Estimation of Point of Contact in Circular Hands

The basic idea behind our work is to use haptic information from the robot to infer (at least in part) environmental kinematics, such as object position and the orientation of the object being manipulated. In the event that this information is already available, e.g., intermittently from cameras, it is beneficial to fusing such data streams. Although this could be seen as merely algebraic changing of variables, we found the geometric approaches such as those based on wrench axis [23,24] especially insightful for manipulation purposes.

**Figure 1:**
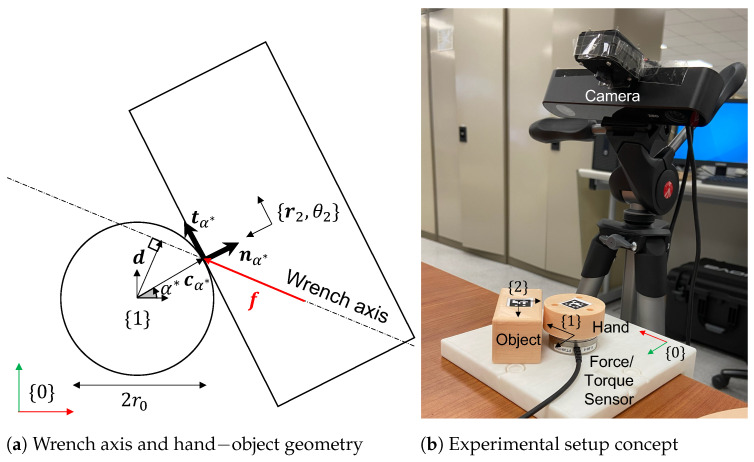
(**a**) Any point on the wrench axis has the same displacement vector d from frame 1. Using Equation (4), we determine $c_{\alpha^{*}}$, the point of pushing. (**b**) This experimental setup was designed to test the proposed method of estimating the point of contact for circular hands.

Many contact tasks of interest involve only forces (not moments) at the point of contact. A moment at the point of contact might arise, for example, from twisting the robotic finger/hand about the normal axis of contact, in the presence of dry friction, but we shall exclude this type of manipulation. Moreover, this eventually can only appear in 3D manipulation while this paper focuses on 2D, planar scenarios. However, moments will be generated at frames located away from the contact point. Typically, force/torque sensing units (loadcells) are located at the wrist of the robot, i.e., away from the end-tip where the contact with the environment occurs. Given a force f at the finger-tip, the moment is determined as:

(1) τ=d×f

where d denotes the displacement vector between the point of contact and the origin of the loadcell frame. Therefore, while the force reading from the loadcell provides directly the components of the applied force, the torque readings provide additional information on where the force is applied. Specifically, given force and torque readings f and τ, the point of application lies on the axis (denoted ‘wrench-axis’) characterized, in 3D space, by the linear equations in (1). In 2D space, the cross product has no meaning, and the wrench axis equation simply becomes:

(2) τ=det([df])

where [df] is the 2×2 matrix with columns $d={\left[d_{x}\;d_{y}\right]}^{T}$ and $f={\left[f_{x}\;f_{y}\right]}^{T}$ (see Figure 1a).

Given the geometry of the object on which a force is being applied, the geometric intersection with the wrench axis ultimately defines the point of contact. In summary, haptic measurements from the robot hand/fingers can be used to infer the point of contact. Additional information such as the geometry of the robotic hand will allow one to determine further variables, such as the normal and tangent vectors at contact, as shown next.

### 2.1. Contact Frame Estimation from Wrench Axis

In the 2D case, the boundary of a solid object is simply a 2D curve. The boundary of a circular hand can be conveniently parameterized by contact parameter α. Any point on the boundary, $c_{\alpha }$ is defined as a function of α and hand radius $r_{0}$. With reference to Figure 1a, when a force is acting at a point $c_{\alpha }$, the wrench axis equation, Equation (2), can also be expressed as:

(3) $$\begin{array}{c} \tau =\det\left(\left[c_{\alpha }\;f\right]\right)=r_{0}\left(\cos\left(\alpha \right)f_{y}-\sin\left(\alpha \right)f_{x}\right) \end{array}$$

Expanding this, we next estimate the parameter $\alpha^{*}$ obtained from haptic feedback in circular hands as:

(4) $$\begin{array}{c} \alpha^{*}\left(f,\tau \right):=-\mathrm{acos}\left(\frac{\tau }{r_{0}\sqrt{f_{x}^{2}+f_{y}^{2}}}\right)+\mathrm{atan}2\left(-f_{x},f_{y}\right) \end{array}$$

After the estimation of $\alpha^{*}$, the contact point and frame may be computed as:

(5) $$\begin{array}{c} c_{\alpha^{*}}=r_{0}n_{\alpha^{*}},\;n_{\alpha }=\left[\begin{array}{c} \cos(\alpha )\\ \sin(\alpha ) \end{array}\right]\;t_{\alpha }=\left[\begin{array}{c} -\sin(\alpha )\\ \cos(\alpha ) \end{array}\right] \end{array}$$

where $n_{\alpha^{*}}$ and $t_{\alpha^{*}}$ represent the normal and tangent at the contact point $c_{\alpha^{*}}$.

### 2.2. Experimental Validation of Estimated Point of Contact

With reference to Figure 1a, given the point of contact at $c_{\alpha^{*}}$ obtained using $\alpha^{*}$ defined above, in our simplified case, we observe that the tangent $t_{\alpha^{*}}$ is aligned with the frame {2}, $t_{\alpha^{*}}\;\left|\right|\;n\left(\theta_{2}\right)$, i.e.,

(6) $$\begin{array}{c} \theta_{2}=\mathrm{atan}2\left(t_{x},t_{y}\right) \end{array}$$

**Figure 2:**
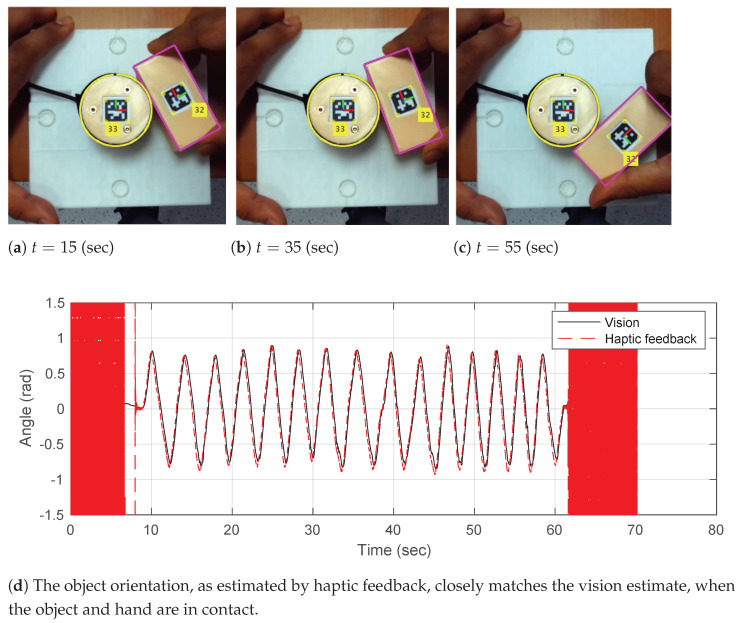
(**a**–**c**) Hand−object configurations during the experiments along with annotated Apriltags for computing the visual estimate of hand−object relative orientation. (**d**) Rolling contact without slipping was ensured by rubber cladding of the hand. Hand−object configurations were varied manually. Object orientation $\theta_{2}$ estimated from Apriltags, i.e., vision, plotted against the orientation computed by haptic feedback using Equations (4) and (6).

To validate that the computation of $\alpha^{*}\left(f,\tau \right)$ is accurate, we conducted an experimental trial where a rectangular object was rolled along the circular hand’s boundary without slipping manually, as shown in Figure 2. The object orientation $\theta_{2}$ was obtained from two sources:

- Vision: using Apriltags, as shown in Figure 2.
- Haptics: using the proposed haptic method $\alpha^{*}\left(f,\tau \right)$ in Equation (4).

Note that in Figure 2, during the initial and final phases where there is no contact, the object orientation, as estimated from haptic feedback, is not well defined. Once rolling without slipping is ensured, the haptic-estimated object orientation follows the visual estimate very closely.

With these initial results, we proceed to define the larger problem of using a dual-arm robotic system with circular hands to determine the object pose, i.e., both position and orientation during dexterous manipulation of a planar object in the next section.

## 3. Kinematics of Planar Dual-Arm Manipulator

**Figure 3:**
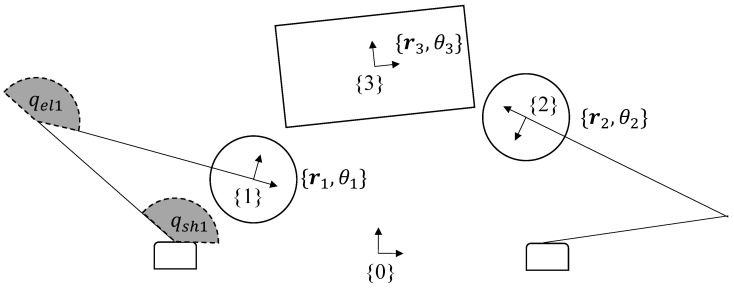
Dual−arm robot kinematic variables.

The planar kinematics (see Figure 3) of the object and the dual-arm robot can be described in terms of rigid planar transformations to and from the following frames:

- Common frame {0}, a fixed frame attached to the work-space (e.g., the table on which the robot is operating);
- Left-hand frame {1}, a moving frame attached to the end-effector of robot arm 1;
- Right-hand frame {2}, a moving frame attached to the end-effector of robot arm 2;
- Object frame {3}, a moving frame attached to the object to be manipulated.

For each frame, we define its location $r\in R^{2}$ and its orientation θ w.r.t the common frame {0}, to be composed as a 3-tuple {r,θ}. To compose the transformation of the local coordinates for, say, robot arm 1 at time *t*, the associated SE(2) transformation denoted by $\Phi_{1t}={\left[r_{1},\theta_{1}\right]}_{SE2}{|}_{t}$ is defined as:

(7) $$\begin{array}{c} \Phi_{1t}=\left[\begin{array}{cc} R(\theta_{1}\left(t\right)) & r_{1}\left(t\right)\\ 0 & 1 \end{array}\right]\;\;\;R\left(\theta \right)=\left[\begin{array}{cc} \cos(\theta ) & -\sin(\theta )\\ \sin(\theta ) & \cos(\theta ) \end{array}\right] \end{array}$$

Similar definitions may be made for the other frames at different times.

The task space coordinates of the end-effector frames defined above are related to the joint variables by the robot’s forward kinematics as:

(8) $$\begin{array}{c} r_{1}=\left[\begin{array}{c} l_{1}\cos\left(q_{sh1}\right)+l_{2}\cos\left(q_{sh1}+q_{el1}\right)\\ l_{1}\sin\left(q_{sh1}\right)+l_{2}\sin\left(q_{sh1}+q_{el1}\right) \end{array}\right]\;\theta_{1}=q_{sh1}+q_{el1} \end{array}$$

Similar computations were made for the second robot arm.

### Hand−Object Surface Parameterization

**Figure 4:**
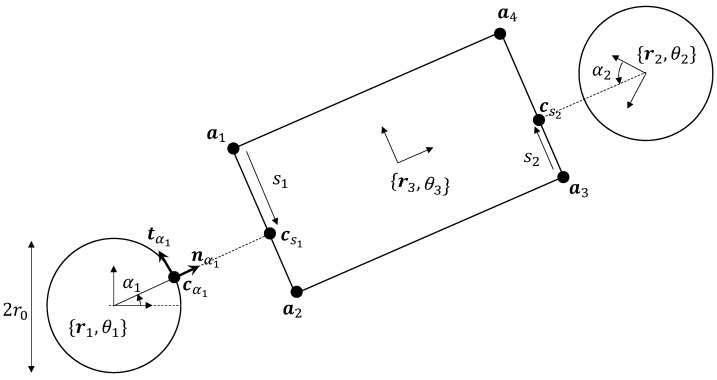
Parameterized surface proxies [25] are virtual points defined as being constrained to be on the surface of objects. By introducing attractive dynamics between these proxies, they can be made to act as the closest points (to the robot hand) on their respective surfaces. In the context of physically interacting objects, the kinematics of such proxies on both surfaces would capture the contact constraints. For instance, with rolling contact between side $a_{1}a_{2}$ and hand 1, the proxies $c_{\alpha_{1}}$ and $c_{s_{1}}$ on two rolling bodies would be coincident with no relative velocity. Similarly, the proxies for hand 2 would follow. We define the notation such that, for each point $c_{\alpha_{h}}$ on hand *h*, the closest point on the object is defined as $c_{s_{h}}$.

As defined before in Section 2.1, any point on the robot hand h={1,2} with parameter $\alpha_{h}$ and radius $r_{0}$ (see Figure 4) can be expressed in local-coordinates as:

(9) $$\begin{array}{c} c\left(\alpha_{h}\right)=r_{0}n_{\alpha_{h}} \end{array}$$

Note that $n_{\alpha_{h}}$ and $t_{\alpha_{h}}$ represent the normal and tangent at the point parameterized by $\alpha_{h}$ expressed in local coordinates. We next define the parameter $s_{1}$ for any point on the side $a_{1}a_{2}$ of the object expressible as:

(10) $$\begin{array}{c} c\left(s_{1}\right)=a_{1}+s_{1}\hat{a_{12}},\;a_{12}=a_{2}-a_{1}\;\hat{a}=\frac{a}{\left||a|\right|} \end{array}$$

Similar definitions can be made for robot hand 2 interacting with side $a_{3}a_{4}$. These definitions will become important when we discuss the rolling contact constraints between the robot hands and their respective object sides being contacted during manipulation, in the next section.

## 4. Haptic-Based Tracking of Object Pose

**Figure 5:**
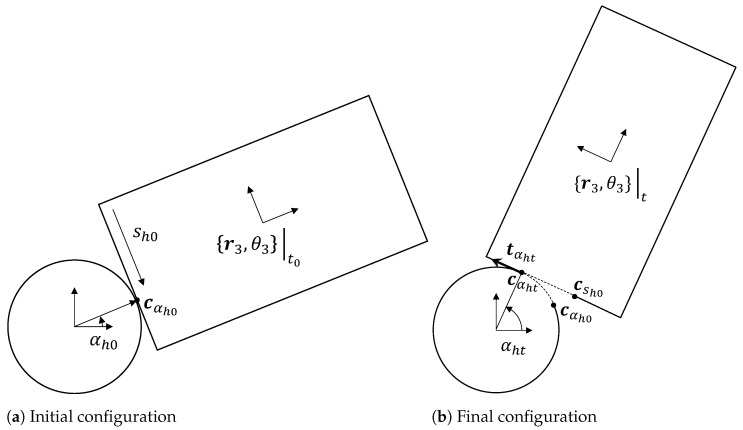
(**a**) Definition of initial hand−object configuration parameterized by $\alpha_{h0}=\alpha^{*}\left(t_{0}\right)$. (**b**) At time *t*, the object position and orientation can be obtained from $\Delta \alpha_{h}=\alpha_{ht}-\alpha_{h0}$. This follows from the rolling without slipping constraint that the distance traversed between $c_{\alpha_{h0}}$ and $c_{\alpha_{ht}}$ (along the hand boundary) should be equal and opposite to that traversed on the object boundary, i.e., from $c_{s_{h0}}$ to $c_{s_{ht}}$.

Under the assumptions of rolling with no slipping defined in the previous subsection, we studied two hand−object configurations obtained via rolling contact, as shown in Figure 5a,b. The initial hand−object configuration is defined by the local geometry at the point of contact, which is parameterized by $\alpha_{h0}=\alpha^{*}\left(t_{0}\right)$ with the normal and tangent in local coordinates also defined as per Equation (5). Similarly, the final configuration was parameterized by $\alpha_{ht}$. The decomposition of relative motion from the initial to final configuration is shown in Figure 5a,b. The following algorithm summarizes these steps to calculate final object pose given the initial pose and wrench information at any time *t*.

It follows that the displacement of the point of contact on the robot hand is parameterized by $\Delta \alpha_{h}=\left(\alpha_{ht}-\alpha_{h0}\right)$ and is given by $r_{0}\Delta \alpha_{h}$, for this case of circular hands. We can also infer that the displacement of point of contact on the object is equal and opposite—i.e., a positive value of $\Delta \alpha_{h}$ will imply a negative value of $\Delta s_{h}$. In other words, since the object has straight sides, we have $\left|\right|c_{s_{ht}}-c_{s_{h0}}\left|\right|=\left|\right|r_{0}\Delta \alpha_{h}\left|\right|$ (see Figure 5b). In summary, with the hand and object always in rolling contact without slipping or break of contact, at any instantaneous point in time, the relative movement of the object w.r.t. the hand is composed of (i) a pure rotation about the robot hand and (ii) a pure translation about the tangent at the contact point.

### Sensor Fusion of Object Pose from Multiple Robot Arms

Assuming that Gaussian white noise is present in the force sensors, we can compute the propagation of uncertainty through Algorithm 1 to obtain $\tilde{r_{3}}$, $\Sigma_{r_{3}}$, i.e., the object position estimate and associated uncertainty, respectively, and $\tilde{\theta_{3}}$, $\sigma_{\theta_{3}}^{2}$, i.e., the object orientation estimate and associated uncertainty, respectively. We define the covariance matrix for the 2D wrenches $\Sigma_{W_{h}}$ (see Appendix A.2 for details) sensed on hand *h* (in local coordinates) as:

(11) $$\begin{array}{c} \Sigma_{W_{h}}=\left[\begin{array}{cc} \Sigma_{f} & \sigma_{f,\tau_{h}}^{2}\\ \sigma_{f,\tau_{h}}^{2} & \sigma_{\tau_{h}}^{2} \end{array}\right],\;W_{h}=\left[\begin{array}{c} f_{h}^{\prime }\\ \tau_{h}^{\prime } \end{array}\right] \end{array}$$

Note that $\Sigma_{W_{h}}$ is a symmetric matrix obtained from sensor data and denotes the sensitivity of the force/torque sensor’s strain gauges; i.e., the more sensitive or accurate they are, the lower the determinant of $\Sigma_{W_{h}}$.
**Algorithm 1** Computing final configuration from (i) knowledge of initial configuration and (ii) wrench in final configuration using information from hand *h*.**Require:**$\alpha_{h0}$, $\left[r_{3},\theta_{3}\right]{|}_{t_{0}}$, $\left[r_{h},\theta_{h}\right]{|}_{t_{0},t}$ and $\left[f_{h}^{\prime },\tau_{h}^{\prime }\right]{|}_{t}$
▹ (’) denotes local frame measurements 
$\alpha_{ht}\leftarrow \alpha^{*}\left(\left[f_{h}^{\prime },\tau_{h}^{\prime }\right]{|}_{t}\right)$
 
$\Delta \alpha_{h}\leftarrow \alpha_{ht}-\alpha_{h0}$
 
$Rot\leftarrow \left[\begin{array}{cc} R(\Delta \alpha_{h}) & 0\\ 0 & 1 \end{array}\right]$▹ Rotation about hand 
$Trn\leftarrow \left[\begin{array}{cc} I_{2} & -r_{0}\Delta \alpha_{h}\;t^{\prime }\left(\alpha_{ht}\right)\\ 0 & 1 \end{array}\right]$    ▹ Back-shifting along tangent 
$\Phi_{30}\leftarrow {\left[r_{3},\theta_{3}\right]}_{SE2}{{|}_{t_{0}},\;\;\Phi_{h0}\leftarrow {\left[r_{h},\theta_{h}\right]}_{SE2}|}_{t_{0}}$      ▹ Initial state 
$\Phi_{30}^{\prime }\leftarrow \Phi_{h0}^{-1}\cdot \Phi_{30}$
 
$\Phi_{3t}^{\prime }\leftarrow Trn\cdot Rot\cdot \Phi_{30}^{\prime }$
       ▹ Final state 
$\Phi_{ht}\leftarrow {\left[r_{h},\theta_{h}\right]}_{SE2}{|}_{t}$
 
$\Phi_{3t}\leftarrow \Phi_{ht}\cdot \Phi_{3t}^{\prime }$

   **return** $\left[r_{3},\theta_{3}\right]{|}_{t}$



Recall that the object position and orientation $\left[r_{3},\theta_{3}\right]{|}_{t}$ at time *t* are the outputs of Algorithm 1 with the input parameters $\alpha_{h0}$, $\left[r_{3},\theta_{3}\right]{|}_{t_{0}}$, $\left[r_{h},\theta_{h}\right]{|}_{t_{0},t}$ and $\left[f_{h}^{\prime },\tau_{h}^{\prime }\right]{|}_{t}$. To distinguish the noisy object pose information coming from each hand *h*, we re-define the outputs of the algorithm at time *t* as $\left[r_{3,h},\theta_{3,h}\right]{|}_{t}$. To obtain these estimates, we define the Jacobian of our algorithm as:

(12) $$\begin{array}{c} J_{W_{h}}=\nabla_{W_{h}}\left[\begin{array}{c} r_{3,h}\\ \theta_{3,h} \end{array}\right] \end{array}$$

We decompose the Jacobian $J_{W_{h}}$ to determine the covariances $\Sigma_{r_{3}}$ and $\sigma_{\theta_{3}}^{2}$ estimated by one hand *h*, defined as follows:

(13) $$\begin{array}{c} \Sigma_{r_{3,h}}=J_{r_{3}}\Sigma_{W_{h}}J_{r_{3}}^{T}\;\sigma_{\theta_{3,h}}^{2}=J_{\theta_{3}}\Sigma_{W_{h}}J_{\theta_{3}}^{T}\;J_{W_{h}}={\left[\begin{array}{c} J_{r_{3}}\\ J_{\theta_{3}} \end{array}\right]}_{3\times 3} \end{array}$$

Given multiple hands, one can fuse these probability distributions from each hand *h* assuming independence, by the product of Gaussians [26]:

(14) $$\begin{array}{c} \Sigma_{r_{3}}={\left(\Sigma_{r_{3,1}}^{-1}+\Sigma_{r_{3,2}}^{-1}\right)}^{-1}\;\tilde{r_{3}}=\Sigma_{r_{3}}\left(\Sigma_{r_{3,1}}^{-1}r_{3,1}+\Sigma_{r_{3,2}}^{-1}r_{3,2}\right) \end{array}$$

Similar equations follow to estimate the object orientation as well. We now have all the tools required to compute the estimate for object position and orientation from two hands and their kinematic and haptic feedback. In the next section, we validate our method with ou experimentation.

## 5. Object-Pose Estimation Results

**Figure 6:**
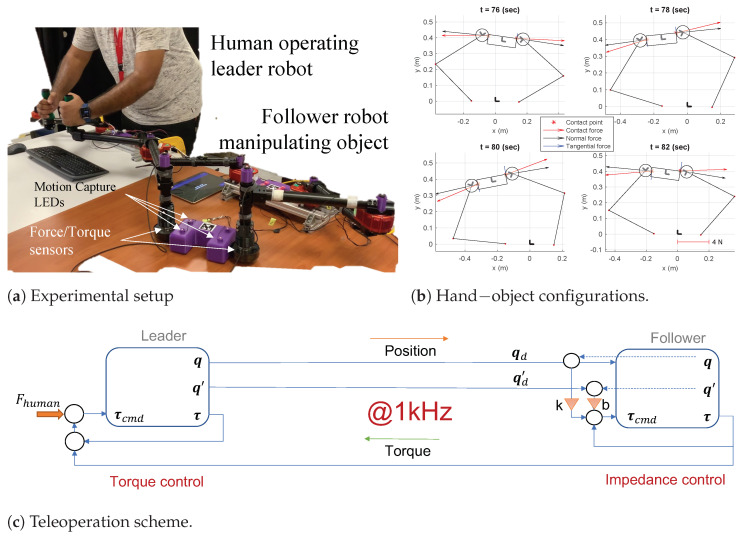
(**a**) Experimental setup with motion-capture LEDs mounted for kinematic tracking and the robot hands equipped with force/torque sensors to sense haptic feedback during manipulation. (**b**) Hand−object configurations and equilibrium forces during the experiment. The decomposition of the equilibrium force into normal and tangential components at the contact point is also shown. (**c**) Impedance−control−based bilateral teleoperation scheme for dual-arm manipulation human haptic demonstrations.

To test the proposed approach of estimating object pose in planar dual-arm manipulation, a robotic test-bed was built, as shown in Figure 6a. Since the scope of this work does not include grasp synthesis and planning, we used human haptic demonstrations of dexterous manipulation performed using a teleoperated dual-arm robot. This choice was in pursuit of a larger goal to understand human haptic strategies in dual-arm manipulation, although it is beyond the scope of this work. A key advantage of our approach to estimating the contact frame is the possibility of reconstructing the normal and tangential forces at contact (see Figure 6b). This adds on to previous research [12] that heuristically set normal forces to control the object pose in simulation. With our approach, we provide an experimental way to generate data from human haptic demonstrations of these internal forces of grasping. Note that although we show results for circular hands, our approach may be extended to any parametric 2D curve that the hand may be shaped as. The teleoperation scheme was designed with consideration for haptic transparency [27] (see Figure 6c), which allows for the impedance control of the robots.

Object kinematics $\left[r_{3},\theta_{3}\right]{|}_{t}$ were determined using a motion capture system that tracks the LEDs mounted on the object (see Figure 6a). The motion capture system was also calibrated with the robot joint feedback to obtain robot-hand kinematics $\left[r_{h},\theta_{h}\right]{|}_{t}$ for h={1,2}. We utilized an oracle to guide the calibration procedure and ensure the object kinematics were consistent with the robot kinematics obtained using on-board sensors. The detailed approach is presented in [28], which was also used in this work with the oracle replaced by a highly accurate motion tracking system, i.e., PTI Phoenix Visualeyez. Furthermore, calibrated wrenches $W_{cal}$ (sensed by the force/torque sensors) are used as $W_{h}=W_{cal}$ (computing $W_{cal}$ from sensed wrenches is discussed in Appendix A.1). These form the inputs to the Algorithm 1.

**Figure 7:**
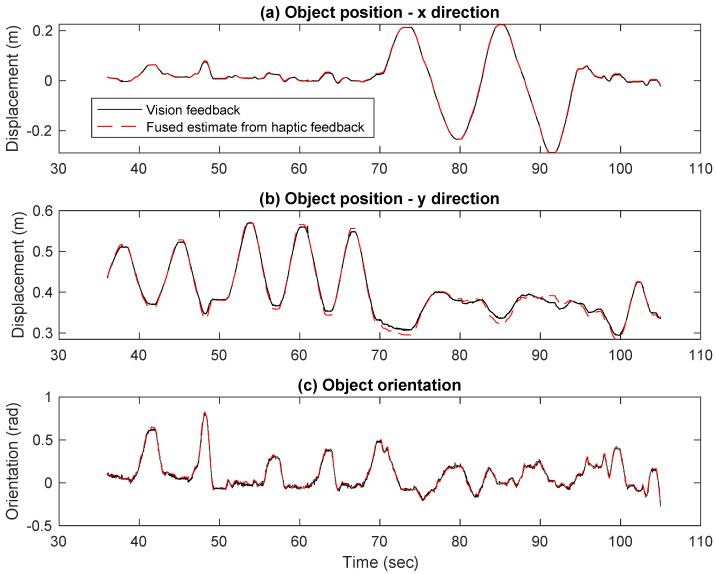
(**a**) Object position in x direction. (**b**) Object position in y direction. (**c**) Object orientation. All measurements are reported in frame {0}.

We selected an initial configuration where the slave robot established a firm grasp on the object at $t_{0}$ = 36 s, which also set $\alpha_{h0}$. Algorithm 1 was evaluated for all time instances in the experiment given this initial condition. To evaluate our estimates, we propagated the wrench uncertainty through Algorithm 1. First, the force/torque sensors were kept static under no load conditions, and readings were taken to compute $\Sigma_{W_{h}}=\mathrm{cov}\left(W_{h}\right)$, as detailed in Appendix A.2. Using Equations (11)−(14), we then estimated the position and orientation, i.e., $\tilde{r_{3}}$ and $\tilde{\theta_{3}}$ estimated at each time instant. The haptic estimated object position and orientation closely follow the motion tracking results, as shown in Figure 7. This validates the proposed method.

### Discussion

Among recent works investigating in-hand/dual-arm manipulation for object handling and object-pose estimation, there is a mix of approaches dealing with known and unknown objects. Broadly, these may be classified into global and local methods; each work focused on a different aspect of dexterous manipulation. Among promising recent works in global sensor fusion of tactile information with vision for in-hand localization to estimate the object pose was [29], and subsequent impedance control of object pose in [30]. Their approach evaluates (for known objects) the robot kinematics, possible collisions with the object, contact points and forces, along with visual tracking of object features to refine the in-hand object pose. Among the local methods, the field of blind tactile grasping controls the relative object pose (to the initial grasp) using incremental shifts after estimating contact points via control of the relative orientation object axis to the contact normals [31]. The original idea was derived from an earlier work on the dexterous control of a circular object [32]. Blind grasping methods use the definition of a virtual frame, which is a polygon created by the points of contact on multi-fingered hands. These works [17,18,33,34] present the control of the virtual frame using the standard definitions of object−hand kinematics and dynamics in multi-fingered manipulation. However, blind grasping methods typically rely on addition sensing modalities such as vision or tactile sensing to estimate the actual object pose. Our work directly contributes to this requirement by providing an additional method to sense object pose using haptic feedback during rolling contact.

Despite the example presented in this work being a polyhedral object, the key point to note is to use parameterized curves/surfaces to represent robot and object geometries. This is because robots interact with the world through curves and surfaces. Different representations of geometries have different advantages. The same polyhedral object may be approximated by a differential geometric curve that allows one to define a continuous parameter for every point, which makes it suitable to work with continuous motions that robots make. Our motivation in choosing this approach of utilizing moving-frame method (originally developed in the field of differential geometry) to robots interacting with objects through rolling contact was extensively studied in [35]. In future work, we will study the extension of our method to objects of arbitrary geometry along these lines.

Another consideration when implementing the proposed method is the stability of the teleoperation scheme. In this work, to avoid instability, the robot was moved slowly enough, allowing the human to have a chance to respond to any communication delays. In the event of communication delays, the reader is referred to a recent survey on telerobotic time delay mitigation [36] to take note of the various predictive methods involved in addressing such communication delays.

## 6. Conclusions

In this work, a haptic method for estimating the contact frame on a circular hand was detailed. Experimental validation of the proposed haptic method was first done for one hand by tracking the object orientation of an object under rolling contact. With this proof of concept for one hand, an algorithm to track the object position and orientation with two robot hands was presented. The kinematics of the dual-arm robot were detailed, along with the mathematical formulation of rolling contact constraints in terms of (i) rotation about robot hand and (ii) translation about the contact tangent. The final object pose was computed as a composition of these two transformations. This algorithm can also be extended to multiple hands, along with the possibility of a probabilistic estimate of the object state. An experimental robotic test-bed was built which allows a human to perform the dual-arm manipulation by teleoperation to test our proposed haptic method. The object pose was tracked using a motion capture system with high accuracy and compared to the pose computed using our method, which validated our approach.

## Figures and Tables

**Figure A1:**
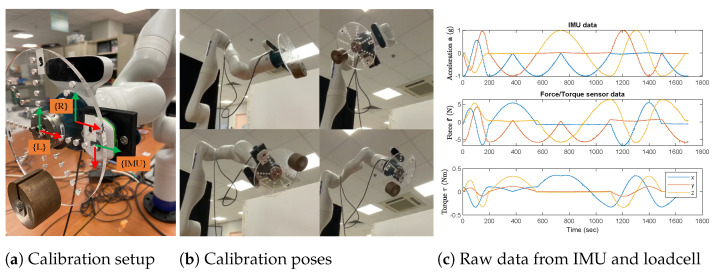
(**a**) The calibration setup consisted of the loadcell. (**b**) The calibration setup was held by a robot at several static poses, as shown, to obtain synchronous data from both sensors (**c**) The IMU data show the true effect of gravity on the calibration rig and was used to compute the expected forces to calibrate the forces recorded by the loadcell, also shown.

**Figure A2:**
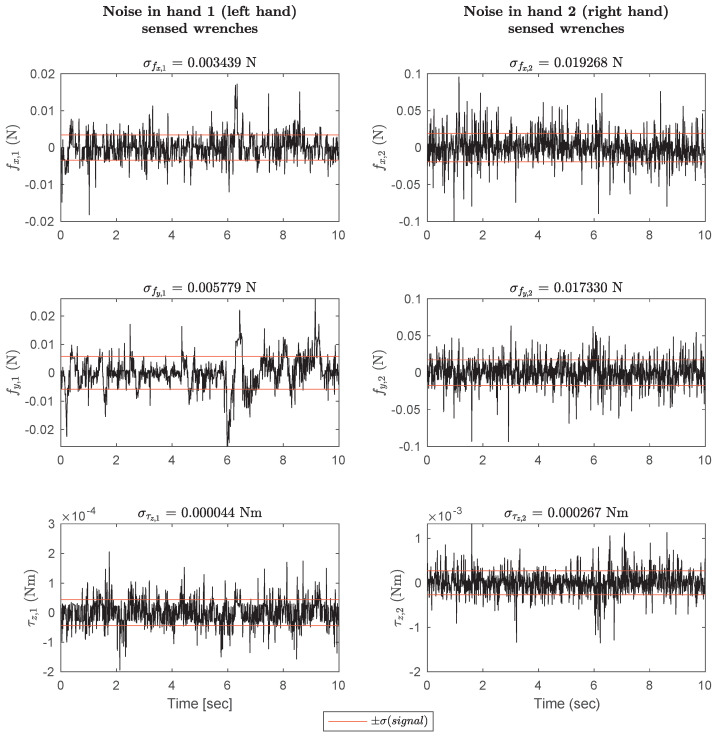
The force/torque sensor on both robot hands was kept static under no-load conditions, and data were collected for 10 s at 1000 Hz. The signals are portrayed here, along with the associated standard deviation per signal computed from the covariance matrix $\Sigma_{W_{h}}$.

## Appendix A

### Appendix A.1. Calibration of Force/Torque Sensors

Before using the force/torque sensors, it is important to determine their error in measurements. This means to compare the (i) measured forces and torques from the sensor given a set of (ii) expected forces and torques. To generate the expected forces, a known calibration mass is mounted on the sensor at a pre-defined location. With knowledge of the kinematics of this calibration mass in the gravity field, one can compute the expected forces. The calibration can then be a least-squares regression between these expected values and the measured ones along with an estimate of the error in the prediction offered by the regression model.

To this end, a calibration setup was built, as shown in Figure A1a, with an IMU and a loadcell in frames {IMU} and {L}, respectively. This setup was held static against gravity using a robot, as shown in Figure A1b, through several poses to obtain a set of expected forces and measured forces. The IMU acceleration measurements $a_{i}\in R^{3}$ allow one to compute the expected forces $\tilde{f_{i}}=ma_{i}$, where the *m* is the calibration mass which was measured on a weighing scale.

Force/torque sensors are typically built as strain gauges [37], with a (i) linear gain term amplifying the deflection of the gauge from (ii) an equilibrium point. Thus, we modeled our force/torque sensor similarly and aimed to regress the gain and the equilibrium bias. To this end, the model we chose to calibrate the forces yielded the following equation for each measurement $f_{i}$:

(A1) $$\begin{array}{c} {\left(\frac{f_{xi}-f_{0x}}{c_{x}}\right)}^{2}+{\left(\frac{f_{yi}-f_{0y}}{c_{y}}\right)}^{2}+{\left(\frac{f_{zi}-f_{0z}}{c_{z}}\right)}^{2}=\left|\right|f_{i}\left|\right| \end{array}$$

Here, the $c={\left[c_{x}\;c_{y}\;c_{z}\right]}^{T}$ is the gain term in the calibration of forces, and the bias term is given by $f_{0}={\left[f_{0x}\;f_{0y}\;f_{0z}\right]}^{T}$, both of which are regressed from calibration data by minimizing the following cost function [38]:

(A2) $$\begin{array}{c} \min_{c,f_{0}}\sqrt{{\left(f_{xi}-f_{0x}\right)}^{2}+{\left(f_{yi}-f_{0y}\right)}^{2}+{\left(f_{zi}-f_{0z}\right)}^{2}}\cdot \left[1-\frac{\left|\right|\tilde{f_{i}}\left|\right|}{{\left(\frac{f_{xi}-f_{0x}}{c_{x}}\right)}^{2}+{\left(\frac{f_{yi}-f_{0y}}{c_{y}}\right)}^{2}+{\left(\frac{f_{zi}-f_{0z}}{c_{z}}\right)}^{2}}\right] \end{array}$$

After these terms were determined using non-linear least squares regression (implemented in MATALB using lsqnonlin), the recalibrated forces per each measurement may be computed as:

(A3) $$\begin{array}{c} f_{CAL,i}={\mathrm{diag}}^{-1}\left(c\right)\cdot \left(f_{i}-f_{0}\right),\;\mathrm{where},\;\;\;{\mathrm{diag}}^{-1}\left(c\right)=\left[\begin{array}{ccc} 1/c_{x} & 0 & 0\\ 0 & 1/c_{y} & 0\\ 0 & 0 & 1/c_{z} \end{array}\right] \end{array}$$

Similarly, expected torques may be computed by $\tilde{\tau_{i}}=r_{COM}\times \tilde{f_{i}}$. For each *i*-th measurement, we have

(A4) $$\begin{array}{c} \tilde{\tau_{i}}=\tau_{0}+\mathrm{diag}\left(g\right)\tau_{i},\;\;\;\mathrm{where},\;\;\mathrm{diag}\left(g\right)=\left[\begin{array}{ccc} g_{x} & 0 & 0\\ 0 & g_{y} & 0\\ 0 & 0 & g_{z} \end{array}\right] \end{array}$$

Here, $\tau_{0}$ is the bias term and g is the gain term. We rewrote Equation (A4) in matrix form to stack up all measurements and regress the bias and gain:

(A5) $$\begin{array}{c} \tilde{\tau_{i}}=A_{i}\left[\begin{array}{c} g\\ \tau_{0} \end{array}\right],\;\;\;\mathrm{where},\;A_{i}=\left[\mathrm{diag}\left(\tau_{i}\right)\;I_{3}\right] \end{array}$$

A least-squares regression estimates both g and $\tau_{0}$, which are used to calculate the re-calibrated torques $\tau_{CAL,i}$. Together with $f_{CAL,i}$, the re-calibrated wrench is composed as:

(A6) $$\begin{array}{c} W_{CAL,i}=\left[\begin{array}{c} f_{CAL,i}\\ \tau_{CAL,i} \end{array}\right]\;\mathrm{where},\;\;\;\tau_{CAL,i}=\tau_{0}+\mathrm{diag}\left(g\right)\tau_{i} \end{array}$$

### Appendix A.2. Noise in Wrenches Sensed on Both Hands

In this work, we used the ATI Mini40 sensor with SI-80-4 calibration specification. According to the datasheet [37], the resolutions offered by the sensor on channels $f_{x}$, $f_{y}$ and $\tau_{z}$ (the sensors used in our experiment) are shown in Table A1:

**Table A1:** Comparison of experimentally obtained standard deviation (SD) with sensor resolution reported in the data-sheet.

Quantity Name	Symbol	Value and Units
Resolution of $f_{x}$	res${}_{f_{x}}$	0.02 N
Resolution of $f_{y}$	res${}_{f_{y}}$	0.02 N
Resolution of $\tau_{z}$	res${}_{\tau_{z}}$	0.0005 Nm
SD of $f_{x}$ for hand 1	$\sigma_{f_{x,1}}$	0.0034393 N
SD of $f_{y}$ for hand 1	$\sigma_{f_{y,1}}$	0.0057792 N
SD of $\tau_{z}$ for hand 1	$\sigma_{\tau_{z,1}}$	4.3925 × 10${}^{-5}$ Nm
SD of $f_{x}$ for hand 2	$\sigma_{f_{x,2}}$	0.019268 N
SD of $f_{y}$ for hand 2	$\sigma_{f_{y,2}}$	0.01733 N
SD of $\tau_{z}$ for hand 2	$\sigma_{\tau_{z,2}}$	0.00026706 Nm

To validate the sensor accuracy, we placed the Mini40 loadcells used on both hands for the dual-arm manipulation under no-load conditions and recorded data from them for 10 s at 1000 Hz. These data are shown in Figure A2 for both sensors. The covariance matrix $W_{h}$ was computed from this. They are reported next to detail the noise levels in individual wrench channels, i.e., $f_{x},f_{y},\tau_{z}$.

The covariance of the force/torque sensor on the left hand is:

(A7) $$\begin{array}{c} \Sigma_{W_{1}}=\left[\begin{array}{ccc} 1.1829\times 10^{-5} & 1.9456\times 10^{-6} & 4.261\times 10^{-9}\\ 1.9456\times 10^{-6} & 3.3399\times 10^{-5} & 6.4659\times 10^{-8}\\ 4.261\times 10^{-9} & 6.4659\times 10^{-8} & 1.9294\times 10^{-9} \end{array}\right] \end{array}$$

The covariance of the force/torque sensor on the right hand is:

(A8) $$\begin{array}{c} \Sigma_{W_{2}}=\left[\begin{array}{ccc} 0.00037124 & 4.1716\times 10^{-5} & -1.7737\times 10^{-6}\\ 4.1716\times 10^{-5} & 0.00030033 & 3.4014\times 10^{-7}\\ -1.7737\times 10^{-6} & 3.4014\times 10^{-7} & 7.1323\times 10^{-8} \end{array}\right] \end{array}$$

The square roots of the diagonal elements of these covariance matrices represent the standard deviation of the sensors, reported in Table A1 and also in Figure A2 (in red). The reported standard deviations in wrench sensing (especially for the right hand) correspond closely to the sensor resolution reported in Table A1. It may also be noted that the left-hand sensor is five times less noisy than the right-hand one, both for forces and torques. We identify the study of uncertainty in kinematics of object pose estimated from the wrench uncertainties as future work.
